# Complete Genome Sequence of Burkholderia cenocepacia Phage Paku

**DOI:** 10.1128/mra.01220-21

**Published:** 2022-03-28

**Authors:** Sefanit Rezene, Guichun Yao, Tram Le, Ben Burrowes, Carlos Gonzalez, Mei Liu, Jason Gill

**Affiliations:** a Department of Biochemistry and Biophysics, Texas A&M University, College Station, Texas, USA; b Center for Phage Technology, Texas A&M University, College Station, Texas, USA; c BB Phage Consultancy, LLC, Georgetown, Texas, USA; Loyola University Chicago

## Abstract

Burkholderia cenocepacia is able to cause infections in cystic fibrosis patients. B. cenocepacia phage Paku has a 42,727-bp genome sharing a phiKMV-like genome arrangement. T7-like tail components were identified in parallel with a tyrosine integrase, suggesting that Paku might exhibit a temperate lifestyle, an atypical feature for an *Autographiviridae* phage.

## ANNOUNCEMENT

Burkholderia cenocepacia is an opportunistic pathogen that is found in the environment and is known to cause infections in cystic fibrosis (CF) patients that are difficult to treat, because of its antibiotic resistance and possession of multiple virulence determinants (1). Research has been conducted to better understand B. cenocepacia as a pathogen, in hopes of increasing CF patients’ life expectancy (2). We are interested in understanding the genomic diversity of B. cenocepacia phages in order to develop phage therapy for controlling this bacterium.

Bacteriophage Paku was isolated from a soil sample obtained in 2018 in Lincoln, Nebraska, using Burkholderia cenocepacia GIIIa as the host. The soil sample was filtered (0.2-μm pore size), plaque purified three times, and propagated on B. cenocepacia using the soft agar overlay method as described previously (3). DNA was purified by the modified Wizard kit protocol described by Summer (4). DNA libraries were prepared with 300-bp inserts with a Swift 2S Turbo kit and sequenced with a MiSeq Nano system using 500-cycle v2 chemistry. The total of 17,386 raw reads were quality controlled using FastQC (www.bioinformatics.babraham.ac.uk/projects/fastqc) and trimmed with the FASTX-Toolkit v0.0.14 (http://hannonlab.cshl.edu/fastx_toolkit). The genome was assembled with SPAdes v3.5.0 (5), resulting in a single contig with 65.0-fold coverage. The genome was closed bioinformatically based on alignment to another contig with a different opening. Annotation was done on the Center for Phage Technology (CPT) Galaxy-Apollo phage annotation platform (https://cpt.tamu.edu/galaxy-pub) (6–8). This process was broken into two parts, with the structural annotation done by GLIMMER v3 (9) and MetaGeneAnnotator v1.0 (10). tRNAs were detected with ARAGORN v2.36 (11) and tRNAScan-SE v2.0 (12). The functional annotation was completed by use of InterProScan v5.48 (13), BLAST v2.9.0 (14), TMHMM v2.0 (15), HHpred (16), LipoP v1.0 (17), and SignalP v5.0 (18). BLAST searching against the NCBI nonredundant and Swiss-Prot databases (19) was utilized. A genome-wide DNA sequence similarity analysis was performed by progressiveMauve v2.4 (20). All tools were run with default settings unless otherwise specified.

Phage Paku has a 42,727-bp genome with a coding density of 95.6% and a GC content of 61.9%. The precise genome termini could not be determined by PhageTerm (21). In total, 55 protein-coding genes and 1 tRNA gene were predicted. NCBI taxonomy has placed Paku in the subfamily *Okabevirinae*; however, it shares only ∼30% nucleotide identity with other members of this subfamily and thus would likely constitute its own genus in this group. Paku shares 31 proteins (BLASTp, E value of <10^−5^) with *Burkholderia* phage Bp-AMP4 (GenBank accession number HG796221) and 30 proteins with *Burkholderia* phages Bp-AMP1, Bp-AMP2, Bp-AMP3, and AMP1 (GenBank accession numbers HG793132, HG796219, HG796220, and MN191861, respectively) and also shares a phiKMV-like genome arrangement in which the phage RNA polymerase is located in the central portion of the genome. Multiple T7-like tail components were identified in Paku, including homologs of the T7 tail fiber protein gp17 and tail tubular protein gp12. The endolysin of Paku contains an N-terminal signal-arrest-release (SAR) sequence, with the spanin complex directly downstream of the endolysin gene. A tyrosine integrase was identified, raising the possibility that Paku might exhibit a temperate lifestyle, an atypical feature for an *Autographiviridae* phage.

### Data availability.

Paku’s genome was deposited in GenBank with accession number MZ326863. The associated BioProject, SRA, and BioSample accession numbers are PRJNA222858, SRR14095249, and SAMN18509701, respectively.
